# Bio-Based Epoxy-Phthalonitrile Resin: Preparation, Characterization, and Properties

**DOI:** 10.3390/molecules29215019

**Published:** 2024-10-24

**Authors:** Yanqin Du, Ruojin Wang, Qingxu Meng, Xiaoa Zhang, Riwei Xu

**Affiliations:** College of Materials Science and Engineering, Beijing University of Chemical Technology, Beijing 100029, China; d1584946123@163.com (Y.D.); ruojin1208@gmail.com (R.W.); mqx2091093720@outlook.com (Q.M.)

**Keywords:** epoxy resin, bio-based, heat resistance

## Abstract

Preparation of high-performance thermosetting resins via bio-based resources is important for the development of a sustainable world. In this work, we proposed the introduction of cyanide structure groups into the molecular structure of epoxy resins to give them excellent heat resistance. A eugenol-based epoxy-phthalonitrile (EEPN) resin was synthesized by a two-step process using the bio-based renewable resource of eugenol, and a series of EEPN/Epoxide resin (E51) blend resins with different EEPN contents were prepared. The structure of the EEPN monomer was characterized and confirmed by Fourier transform infrared (FTIR), nuclear magnetic resonance (NMR), and elemental analysis. The thermal stability and dynamic mechanical properties of the cured resins were investigated by thermogravimetric analysis and dynamic mechanical thermal analysis. The experimental results showed that EEPN had excellent heat resistance; the char yield at 800 °C was 67.9 wt%, which was much higher than that of E51 at 26.3 wt%; and the heat resistance of the blended resins was significantly improved with the increase in the EEPN content.

## 1. Introduction

Thermosetting resins are widely used in aerospace and marine applications due to their high glass transition temperature (*T_g_*), long-term thermal stability, good mechanical properties, and excellent processability. Common thermosetting resins include epoxy and unsaturated polyester resins, bismaleimide, thermoset polyimide, and phthalonitrile (PN), and most of their raw materials come from non-renewable resources [1]. With the growing demand for petroleum products and their increasing negative impact on the environment, polymeric materials from renewable resource sources have received much attention. Studies have shown that bio-based polymers from renewable resources usually have comparable or superior performance characteristics to those of conventional polymeric materials. Therefore, these environmentally friendly polymeric materials are expected to replace petroleum-derived feedstocks, helping to reduce carbon emissions and conserve limited petroleum resources [2,3].

As one of the three major thermosetting resins, epoxy resins (EPs) are widely used in adhesives [4], coatings [5], and high-performance composite matrices [6] due to their high mechanical properties, low curing shrinkage, excellent chemical resistance, and good processability. Nowadays, epoxy resins account for about 70% of the thermoset materials market, of which bisphenol A diglyceryl ether (DGEBA) accounts for about 90% of the global production of epoxy resins, but the use of bisphenol A, the most important raw material, has been restricted in numerous countries and fields due to its biologically low toxicity [7,8]. Therefore, the study of bio-based epoxy resins based on renewable resources is of great environmental and strategic significance to achieve the green and sustainable development of polymer materials. The excellent performance of cured DGEBA is attributed to the rigid structure of its benzene ring. Therefore, bio-based phenols with rigid structural benzene rings have been widely studied as phenolic sources for the synthesis of epoxy resins, such as cashew nut phenol [9,10,11], luteolin [12], 2,5-furofuran carboxylic acid (FDCA) [13,14], magnolol [15], lignin [16,17,18,19], sorbitol [20], isosorbide [21,22], cellulose [23], etc., which have been used for the synthesis of bio-based epoxy resins. Among the many substances that can be used as bio-based feedstocks, eugenol and its derivatives are widely used in the development of high-performance resins such as epoxy derivatives, benzoxazine resins, and bismaleimide resins, due to their compact structures and multifunctional groups [21] (hydroxyl and allyl), as well as their renewability [24] and low toxicity [25], which can be produced from plant sources, and in the design of various functional polymers [26,27,28,29]. Wang et al. [30] prepared a series of multifunctional bio-based epoxy resin precursor TEU-EPs based on eugenol, and the cured product of TEU-EP/DDS had a *T_g_* as high as 207 °C, which had more excellent thermodynamic properties compared with the traditional bisphenol A-based epoxy resin. Miao et al. [28] designed and synthesized a kind of eugenol containing furan ring-based epoxy resin precursor EUFU-EP and used methylhexahydrophthalic anhydride (MHHPA) as the curing agent. The *T_g_* of the EUFU-EP/MHHPA was slightly higher than that of the DGEBA/MHHPA compared with the conventional DGEBA epoxy resin. This is attributed to the unique molecular structure of EUFU-EP. The EUFU-EP/MHHPA also has higher flexural modulus, storage modulus, and flame retardancy.

Furthermore, epoxy resins, as one of the most widely used polymer materials in the industrial field, have high requirements in terms of heat resistance in special areas such as aerospace and aviation [31]. Therefore, the study of bio-based thermosetting resins with good heat resistance is of great significance and is in line with the trend of continuous development of polymer materials. Phthalonitrile (PN) resin, a compound first synthesized by Keller’s research group in the 1970s, has attracted great attention internationally due to its excellent thermo-oxidative stability and flame retardancy and the absence of small molecules in curing [32,33,34], and it has attracted more and more attention in the application fields of aerospace, electronic encapsulation, and flame-retardant materials [35,36]. In addition, PN is the only resin that meets the U.S. Navy flame standard (MIL-STD-2031) [37,38]. Ning et al. [39] synthesized a novel bio-based PN resin (MEG-PN) based on eugenol derivatives. The main advantages of MEG-PN resin over PN resin are the lower curing temperature (281 °C) and excellent processability. The cured MEG-PN resin showed excellent heat resistance at 448 °C, and its char yield at 800 °C was as high as 75.6%. Sun et al. [40] synthesized two phthalonitrile resins (VPN and IVPN) containing spirocyclic acetal structures by a two-step method using pentaerythritol and renewable vanillin and isovanillin as raw materials. Compared with traditional petroleum-based phthalonitrile resins such as bisphenol A, the cured VPN and IVPN resins have good thermal stability and thermo-mechanical properties, high glass transition temperature (*T_g_*), and excellent processing properties. Wang et al. [41] synthesized two bio-based phthalonitriles (EPN and GPN) based on the reaction of eugenol with lignin derivatives and 4-nitro phthalonitrile, respectively. Their glass transition temperatures were characterized by DSC and DMA tests as 394 °C and 392 °C, respectively, and the char yield of both EPN and GPN was greater than 70% at 800 °C.

**Scheme 1:**
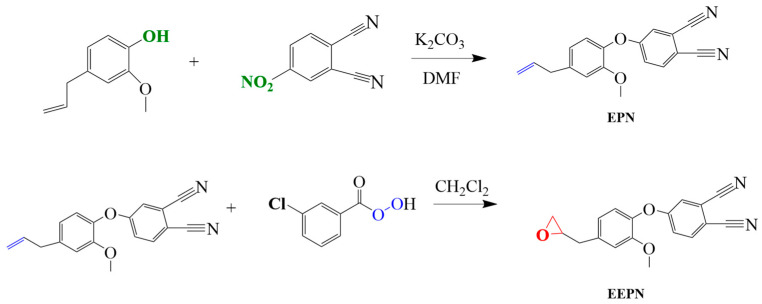
Synthetic routes of EEPN.

In summary, based on the bio-renewable properties of eugenol and the excellent thermo-oxidative stability of PN resins, in this paper, eugenol-based phthalonitrile monomer (EPN) was first prepared from eugenol, o-phenyl 4-nitrophthalonitrile, and potassium carbonate, and then EPN was reacted with 3-chloroperoxybenzoic acid to generate a eugenol-based epoxy-phthalonitrile monomer (EEPN), as shown in Scheme 1. The EEPN was blended with epoxy resin E51. The curing process, curing mechanism, and curing kinetics of EEPN were investigated, and the thermal stability and the dynamic mechanical properties of EEPN and the blended resin were studied.

## 2. Results and Discussion

### 2.1. Characterization of EEPN

**Figure 1:**
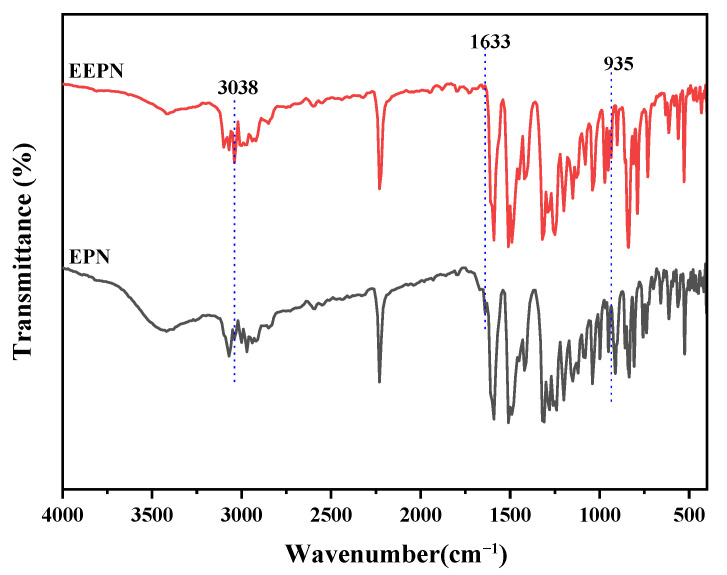
FTIR spectra of EEPN and EPN.

Figure 1 shows the infrared spectra of eugenol-based epoxy-phthalonitrile monomer (EEPN) compared with EPN. From Figure 1, one can observe the disappearance of the allylic stretching vibration peak at 1633 cm^−1^ and the appearance of the asymmetric ring stretching vibration peak of the epoxide ring at 935 cm^−1^, as well as the appearance of the stretching vibration peak of the epoxide ring C–H near 3038 cm^−1^. Thus, it can be indicated that EEPN was successfully synthesized.

**Figure 2:**
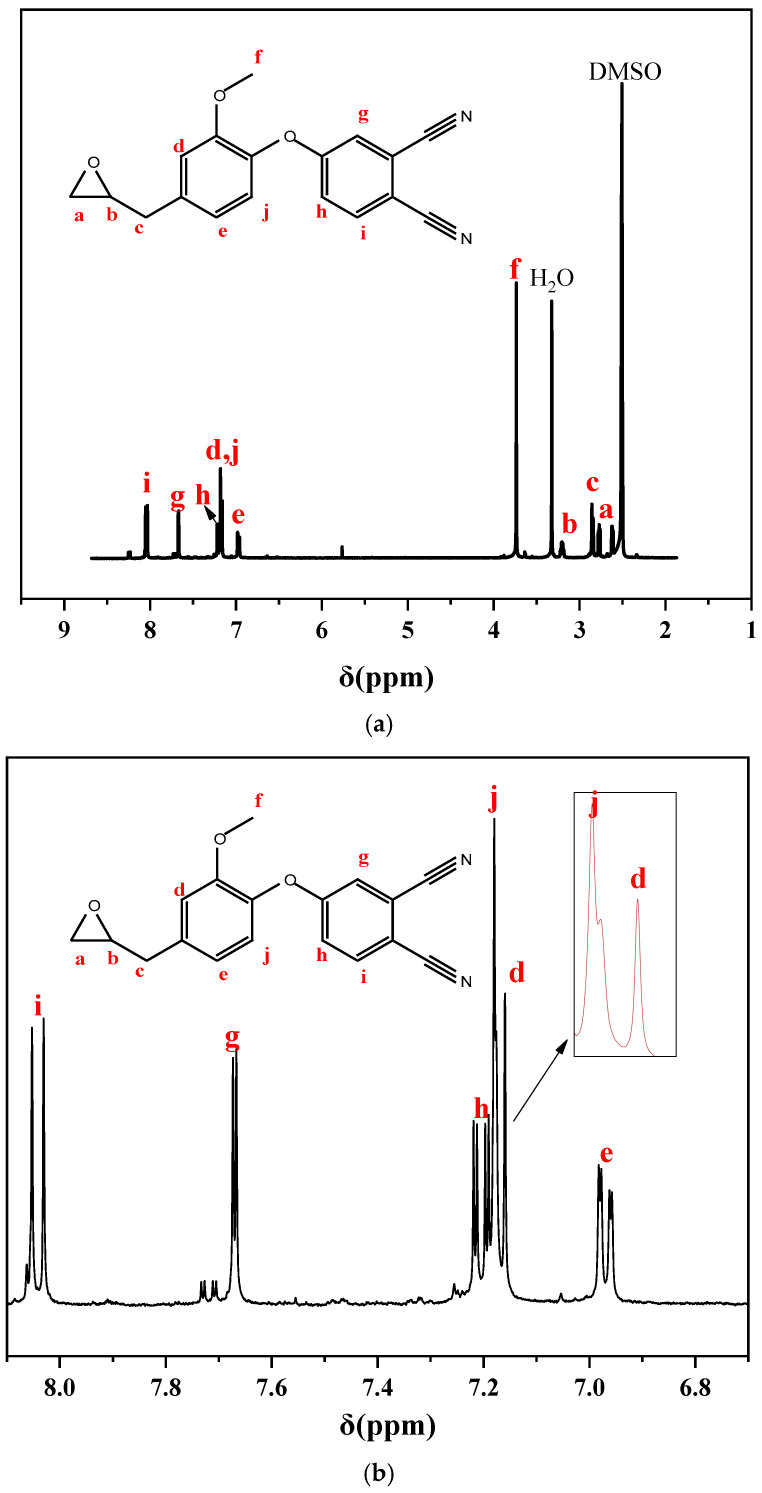
^1^H NMR spectrum of EEPN (**a**) and ^1^H spectrum of EEPN in the range 6.97–8.04 ppm (**b**).

Figure 2a shows the ^1^H NMR spectra of EEPN, which has 10 different chemical shifts of H. From Figure 2, it can be seen that δ = 3.32 ppm is the characteristic absorption peak of H on H_2_O, and 2.50 ppm is the characteristic absorption peak of H on the deuterium-substituted solvent, DMSO. The values δ = 2.62 ppm, δ = 3.20 ppm, and δ = 2.86 ppm are the characteristic peaks on the epoxy groups H_a_, H_b_, and H_c_, respectively; the three hydrogen protons in the methoxy OCH_3_ are H_f_ with a chemical shift of δ = 3.74 ppm; and their integral area ratios are in accordance with the theoretical ratio of 2:1:2:3. The benzene ring structure is a spatially asymmetric three-dimensional structure, so its hydrogen atoms are in different chemical environments, and the aromatic protons of the spectra are confirmed at 6.97–8.04 ppm. We zoomed in the range of 6.97–8.04 ppm. From Figure 2b, it can be seen that H_e_ and H_j_ are coupled to each other and split into double peaks. At the same time, H_e_ is coupled by the interstitial H_d_, and there is a secondary split peak. To better show the peaks out of H_j_ and H_d_, we zoomed in on this part, as shown by the small red peaks in Figure 2b. H_h_ is coupled by the neighboring H_i_ and splits into double peaks, and at the same time, it is coupled by the smaller interstitial of H_g_ and finally shows the morphology of the approximate quadruple peaks. There is also a case of such a mutual coupling between H_g_ and H_i_, and so there is a double peak. The details are as follows: H_d_ (s, δ = 7.16 ppm), H_e_ (d, δ = 6.88–6.86 ppm), H_g_ (s, δ = 7.69 ppm), H_h_ (dd, δ = 7.21–7.19 ppm), H_i_ (d, δ = 8.04–8.02 ppm), and H_j_ (d, δ = 7.18–7.17 ppm), and the ratio of their integral area is 1:1:1:1:1:1, proving that the EEPN is successful on the basis of the combined out EPN.

**Figure 3:**
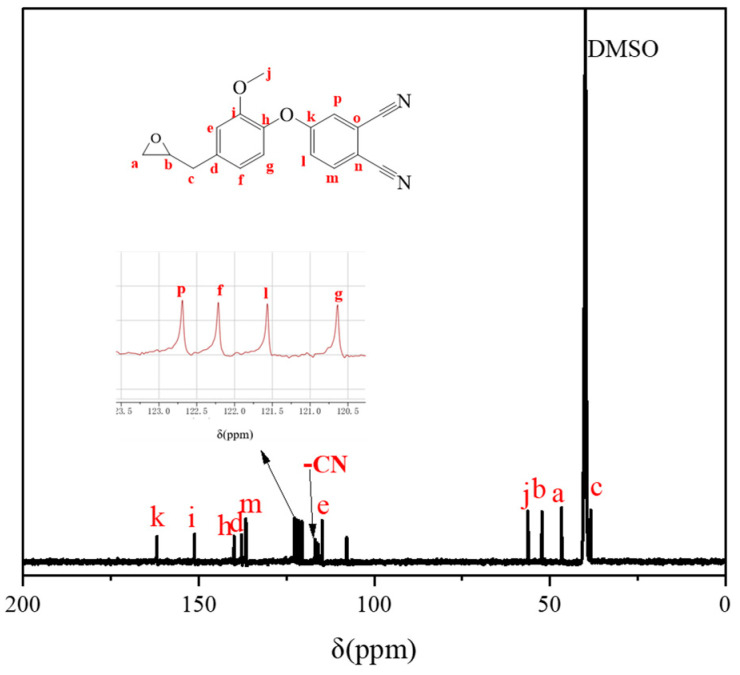
**^13^C** NMR spectrum of EEPN.

The ^13^C NMR spectrum of EEPN is shown in Figure 3. It can be seen that δ = 116.45 ppm and δ = 116.92 ppm are the characteristic peaks of the cyano group, δ = 56.22 ppm are the characteristic peaks of C_j_ for methoxy on the benzene ring, δ = 46.65 ppm and 52.31 ppm are the characteristic peaks of C_a_ and C_b_ for the epoxy group, and δ = 38.41 ppm is the characteristic peak of C_c_. The distribution of aromatic carbon atoms was as follows: C_e_, δ = 114.86 ppm; C_d_, δ = 137.82 ppm; C_f_, δ = 122.22 ppm; C_g_, δ = 120.64 ppm; C_h_, δ = 139.95 ppm; C_i_, δ = 151.22 ppm; C_k_, δ = 161.89 ppm; C_l_, δ = 121.57 ppm; C_m_, δ = 136.6 ppm; C_n_, δ = 107.87 ppm; C_o_, δ = 115.93 ppm; and C_p_, δ = 122.69 ppm. Thus, the structure of EEPN can be further determined.

Table 1 shows the result of elemental analysis of EEPN, which has the molecular formula of C_18_H_14_N_2_O_3_ and a molecular weight of 306.32. The theoretical contents of C, H, and N in EEPN were 70.60%, 4.57%, and 9.15%, respectively, with a theoretical carbon–hydrogen ratio of 15.45, and the actual contents of C, H, and N in the product were 69.69%, 4.43%, and 9.34%, with a theoretical carbon–hydrogen ratio of 15.71. The deviation of C, H, and N contents in the product EEPN was within the error range, indicating that the synthesized EEPN was as expected.

**Figure 4:**
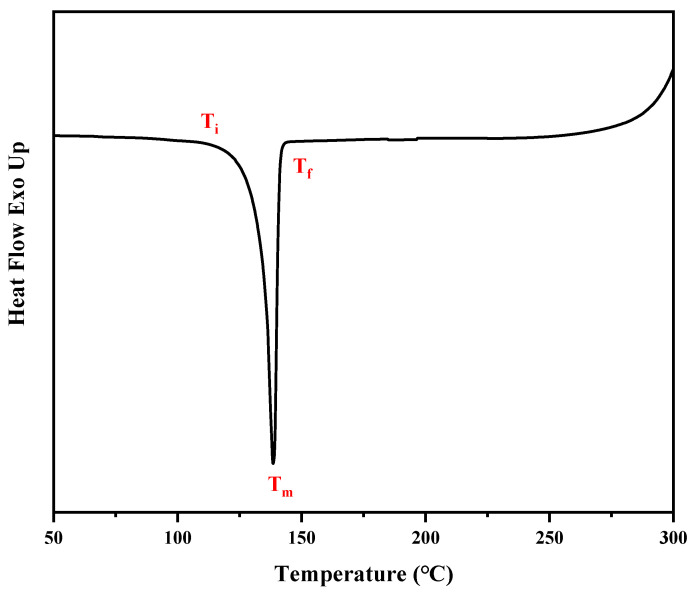
The DSC curve of EEPN.

Figure 4 shows the DSC curve of EEPN, from which it can be seen that the starting melting temperature of EEPN is 125 °C, the melting point is 137 °C, and the termination melting temperature is 142 °C. The enthalpy of melting, ΔH_f_, can be calculated to be 28.7 J/g.

### 2.2. Curing Mechanism for EEPN and EEPN (10%)/E51 Blend Resins Using DDS as Curing Agent via FTIR

The curing mechanism of the amine curing agent DDS curing EEPN and EEPN (10%) blend resins, respectively, was investigated by the analytical method of FTIR spectroscopy as an example of the curing mechanism of the amine curing agent DDS curing EEPN and EEPN/E51 blend resins.

**Figure 5:**
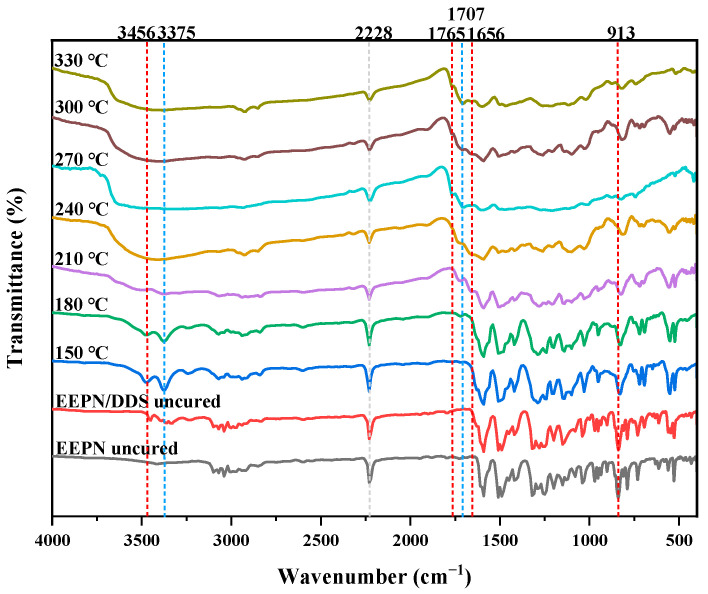
FTIR spectra of stepwise curing of EEPN/DDS. (FTIR samples were obtained from the cure process of EEPN/DDS at 150 °C for 2 h, 180 °C for 2 h, 210 °C for 2 h, 240 °C for 2 h, 270 °C for 2 h, 300 °C for 2 h, and 330 °C for 2 h, and each specific temperature is listed above the curve of the FTIR spectrum).

Figure 5 shows the infrared spectra of the DDS stepwise cured EEPN, from which it can be seen that the characteristic absorption peak of the epoxy group at 913 cm^−1^ in the figure became significantly weaker after warming up and curing compared with the uncured EEPN, indicating that the epoxy group reacted. Meanwhile, the double peaks of primary amines located between 3375 and 3456 cm^−1^ disappeared after elevated temperature curing compared with the uncured EEPN/DDS FTIR spectra plots. In addition, the increase in curing temperature resulted in a significant decrease in the peak intensity of the characteristic absorption peak of the cyano group at 2228 cm^−1^, indicating that the reaction of the cyano group on phthalonitrile occurred. The shift of the cyano absorption peak to a lower wave number suggested the possible formation of triazine derivatives and isoindoline structures. The C=N or C=N–C stretching vibration of the triazine ring was generally in the region of 1600–1700 cm^−1^, and it can be seen from the figure that the characteristic absorption bands of the triazine ring appeared in the range of 1656–1707 cm^−1^ but only after curing at 270 °C. Meanwhile, the characteristic absorption peak of the isoindoline structure around 1765 cm^−1^ appeared only after curing at temperatures above 270 °C.

**Figure 6:**
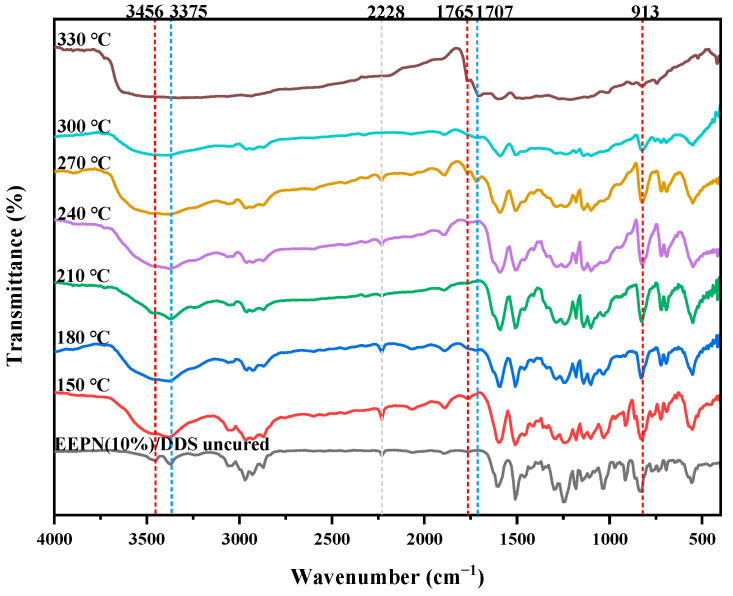
FTIR spectra of stepwise curing of EEPN (10%)/DDS. (FTIR samples were obtained from the cure process of EEPN/DDS at 150 °C for 2 h, 180 °C for 2 h, 210 °C for 2 h, 240 °C for 2 h, 270 °C for 2 h, 300 °C for 2 h, and 330 °C for 2 h, and each specific temperature is listed above the curve of the FTIR spectrum).

Figure 6 shows the infrared spectra of the curing process of EEPN (10%)/DDS at different temperatures. Compared with uncured EEPN (10%)/DDS, it can be seen that the characteristic absorption peaks of the epoxy groups at 913 cm^−1^ became significantly weaker after curing at elevated temperatures, and those of the cyano group at 2228 cm^−1^ became significantly weaker, suggesting that both epoxy groups and the cyano group participated in the polymerization reaction. The infrared spectra proved that there was still unreacted cyano group in the resin when the post-curing temperature was 270 °C. When the post-curing temperature was increased to 330 °C, the cyano group in the EEPN (10%)/DDS resin was basically reacted completely. As can be seen from Figure 6, when the post-curing temperature was below 300 °C, there was still the presence of the cyanide absorption peak located at 2228 cm^−1^ in the EEPN/DDS, indicating that there was still the presence of unreacted cyanide in the resin, and the analysis of the reason may be that the content of the cyanide structure on the less abundant EEPN of the EEPN (10%)/DDS was also relatively small, and it participated in the reaction at high temperatures (higher than 270 °C) to produce triazine derivatives and isoindoline structures more completely.

**Scheme 2:**
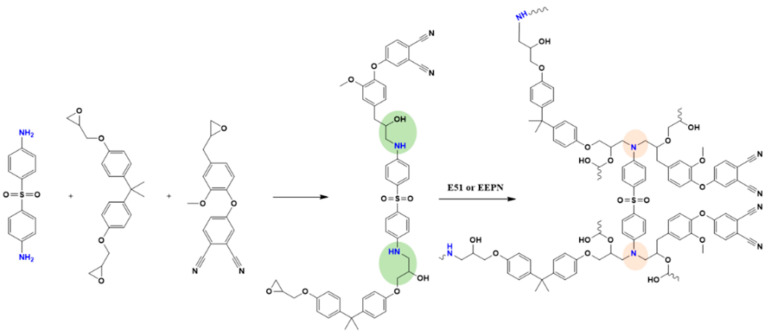

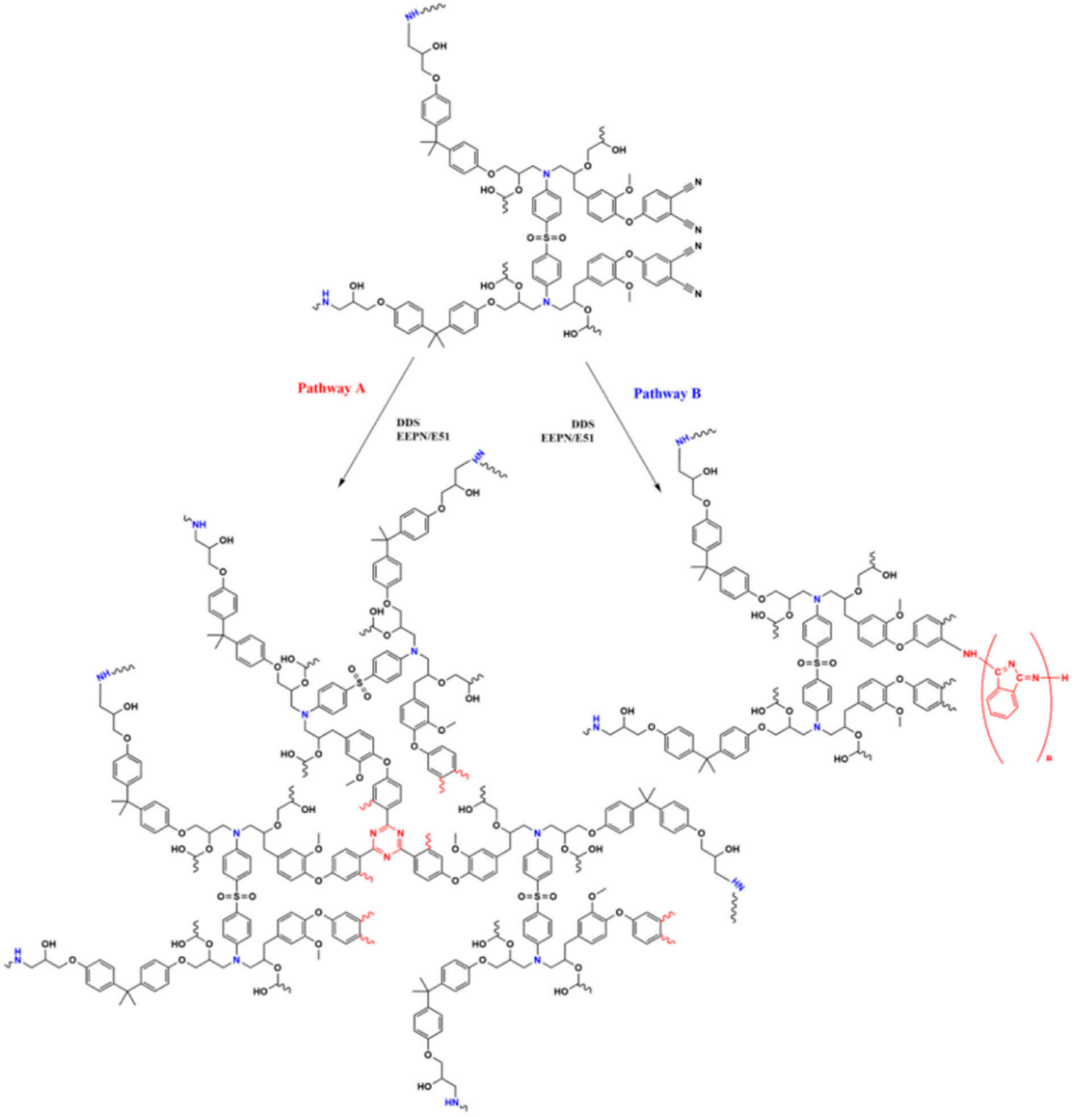
Proposed curing mechanism of EEPN/DDS. (The blue part of the formula indicates the amine group, the green and pink circles mark the binding sites of E51 and EEPN epoxy groups with DDS curing, the red ‘Pathway A’ indicates the route of cyano-curing to form a triazine ring while the blue ‘Pathway B’ indicates the cyano-curing to form an isoindoline structure).

From the analysis based on FTIR spectroscopy as above, it can be surmised that the curing mechanism of the amine curing agent DDS curing EEPN/E51 blend resins is shown in Scheme 2. The main reaction types are the ring-opening reaction between the active hydrogen of the amine group in the amine curing agent and the epoxy group and the nucleophilic addition reaction between the active hydrogen of the amine group and the cyano group. Both the ring opening and nucleophilic addition reactions increased the cross-linking density of the polymer and produced a stable ring structure, which improved the thermal stability of the substance.

### 2.3. Curing Behavior and Curing Kinetics of E51/DDS and EEPN/DDS

In this work, DDS, a commonly used high-temperature amine curing agent, was selected to cure the prepared EEPN/E51 hybrid resins, and a non-isothermal differential scanning calorimetric curve was used to study the curing behavior and curing kinetics of EEPN/DDS. 

**Figure 7:**
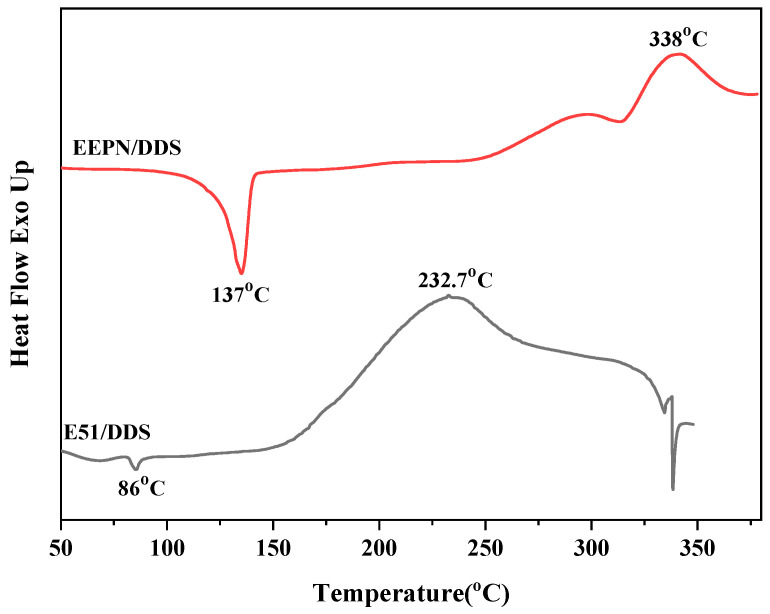
DSC curves of E51/DDS and EEPN/DDS resins.

Figure 7 shows the DSC curves for E51/DDS and EEPN/DDS. As seen in the above graph, the DSC curves of both epoxy resins had a clear exothermic transition, and this exothermic phenomenon was caused by the reaction between the epoxy resin and the curing agent DDS. The onset curing temperature of the E51/DDS was 129.9 °C, the curing exothermic peak temperature of the E51/DDS resin was 232.7 °C, and the processing temperature window (the difference between the onset curing temperature and the exothermic peak temperature) was 102.8 °C. The DSC curve of the EEPN/DDS resin had a clear exothermic transition and a melting peak, and the test showed that the melting point of EEPN was 137 °C, which was close to the melting point of the EEPN monomer as measured by the DSC in the previous section; the cured exothermic peak temperature of this resin was 338 °C, and the processing temperature window was 201 °C.

**Figure 8:**
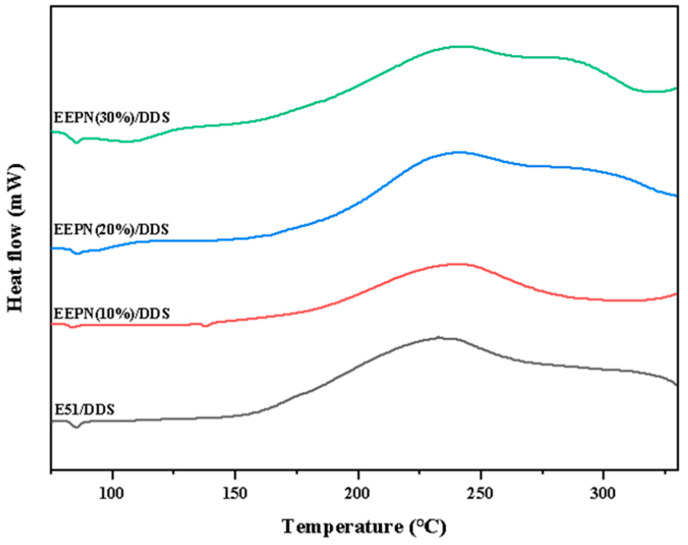
DSC curves of EEPN(n)/DDS resins.

Figure 8 shows the DSC curves of E51/DDS, EEPN (10%)/DDS, EEPN (20%)/DDS, and EEPN (30%)/DDS with a ramp rate of 10 °C/min. Table 2 shows the DSC data of DSC curves of E51/DDS, EEPN (10%)/DDS, EEPN (20%)/DDS, and EEPN (30%)/DDS, where *T_i_*, *T_p_*, *T_f_*, and ∆T are the initial curing temperature, peak curing temperature, end curing temperature, and curing interval, respectively. The analyses in Figure 7 and Figure 8 were performed at a heating rate of 10 °C/min.

As can be seen from Figure 8 and Table 2, the curing temperatures of EEPN (10%)/DDS, EEPN(20%)/DDS, and EEPN (30%)/DDS were higher than that of E51/DDS. The curing intervals were wider, which indicated that the exothermic enthalpy of curing of this hybrid resins was lower and the curing speed was slower, and it required higher curing temperatures and a longer time to cause the EEPN (n)/DDS blend resins to be cured completely.

The curing kinetics of EEPN/DDS were studied through the non-isothermal DSC test performed under different heating rates of 1, 3, 5, and 10 °C/min. The non-isothermal DSC curves of the three EEPN/DDS resins are shown in Figure S4, and the resins exhibited wide curing exothermic peaks at different heating rates. Due to the presence of epoxy groups and cyano groups in the structure of EEPN, two curing peaks did occur during the curing process; the first peak was the curing exothermic peak of the epoxy groups due to their faster exothermicity, and the second one was the curing exothermic peak of the cyano groups. Since this paper mainly investigated the curing process of the cyano group in EEPN in the curing kinetics section, the second peak was taken as the object of study. It can be seen that the exothermic peak of the DSC curve was obvious with the increase in the heating rate, and the exothermic peak was shifted to the high temperature with the increase in the heating rate, while the area gradually increased. This was due to the increase in the rate of the temperature increase; the heat released from the reaction was released in a shorter time, i.e., the reaction heat was released centrally [42].

Figure S5 shows the linear fitting curves for the EEPN/DDS resin. Based on the slopes, we can calculate the apparent activation energy values for the EEPN/DDS resin fitted by the Kissinger and Ozawa equations to be 70.08 kJ/mol and 75.63 kJ/mol, respectively, with a mean activation energy average of 72.85 kJ/mol, which are derived from Tables S4 and S5. The kinetic equation for the curing of EEPN/DDS is shown in Equation (1), which is derived from Table S6.

(1) $$\frac{d\alpha }{dt}=1.066\times 10^{7}exp(\frac{-124.28}{RT}\;)({1-\alpha )}^{1.0138}$$

### 2.4. Thermal Stability

**Figure 9:**
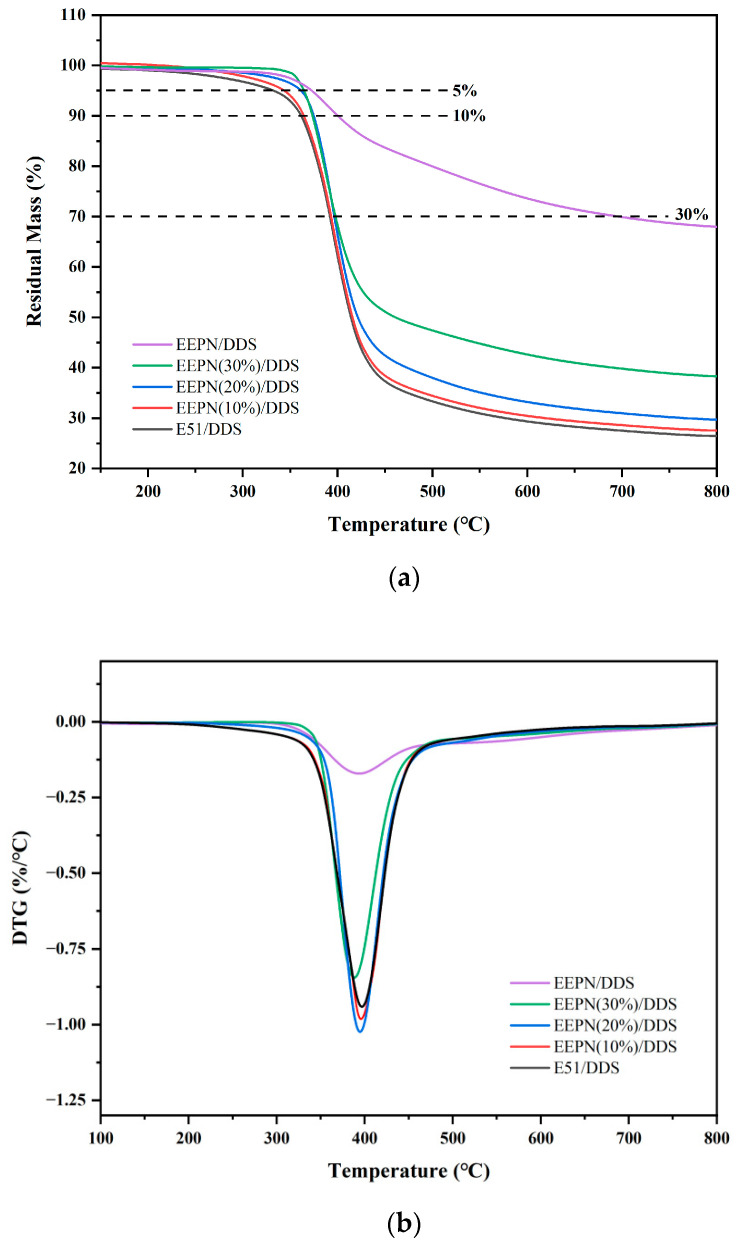
(**a**)TGA and (**b**) DTG curves of E51/DDS and EEPN (n)/DDS (N_2_).

Figure 9 shows the TGA and DTG curves of cured resins for E51/DDS and EEPN(n)/DDS blend resins (n = 10%, 20%, 30%) heated from room temperature to 800 °C in a nitrogen atmosphere.

Table 3 shows the temperatures at 5% weight loss (*T_d5_*), 10% weight loss (*T_d10_*), 30% weight loss (*T_d30_*), maximum thermal weight loss rate temperature *T_d_* max, char yield at 800 °C (*Y_c800_*), and the statistical heat resistance index [43] (*T_s_*) of E51/DDS versus EEPN(n)/DDS in a nitrogen atmosphere, where *T_s_* can be used to qualitatively compare the thermal stability of various resins and can be obtained from Equation (2).

(2) $$T_{s}=0.49(T_{d5}+0.6(T_{d30}-T_{d5}))$$

From the data in Table 3, it can be seen that the *T_d5_* of the EEPN (*T_d5_* = 372.2 °C) was significantly higher compared with the *T_d5_* of the epoxy resin E51 (*T_d5_* = 330.2 °C). It can also be seen that the onset degradation temperature of the resin gradually increased by gradually increasing the content of EEPN in the EEPN/E51 blend resins from 0% to 30%. In terms of the amount of char yield of the cured material, the amount of char yield in the cured material of pure EEPN resin at 800 °C (*Y_c800_*) was 67.9%, which was much higher than that of pure epoxy resin (*Y_c80__0_* = 26.3%). After the introduction of the phthalonitrile group, the char yield at 800 °C of the cured product gradually increased with the increase in the EEPN addition.

In the DTG curve, it can be observed that there was a clear peak in the interval of 310–490 °C for the cured material of E51/DDS resin, the decomposition rate was accelerated from 310 °C, and the maximum value of the decomposition rate was reached at 400 °C. The cured material of EEPN/DDS resin had a clear peak in the interval of 310–460 °C, the decomposition rate was accelerated from 310 °C, and the maximum value of the decomposition rate was reached at 393.7 °C. The decomposition rate was accelerated from 310 °C, and reached the maximum decomposition rate at 393.7 °C. Its thermal decomposition rate was significantly lower than that of the E51/DDS resin.

When the reaction between phthalonitrile and epoxy groups in the eugenol-based epoxy-phthalonitrile resin occurred under the action of the curing agent DDS, the thermal degradation temperature and char yield were higher than that of the E51/DDS resin, and the thermal decomposition rate was lower than that of the E51/DDS resin, which indicated that the thermal performance of the eugenol-based epoxy-phthalonitrile resin synthesized in the present work was superior to that of the traditional bisphenol A-type epoxy resin E51. In addition, according to the thermogravimetric analysis data of the cured materials of EEPN (n)/DDS series hybrid resins, the introduction of the phthalonitrile group could significantly improve the thermal stability of the traditional epoxy resin E51, and the higher the amount of EEPN added, the better the thermal stability of the hybrid resin.

### 2.5. Dynamic Mechanical Properties Studies

**Figure 10:**
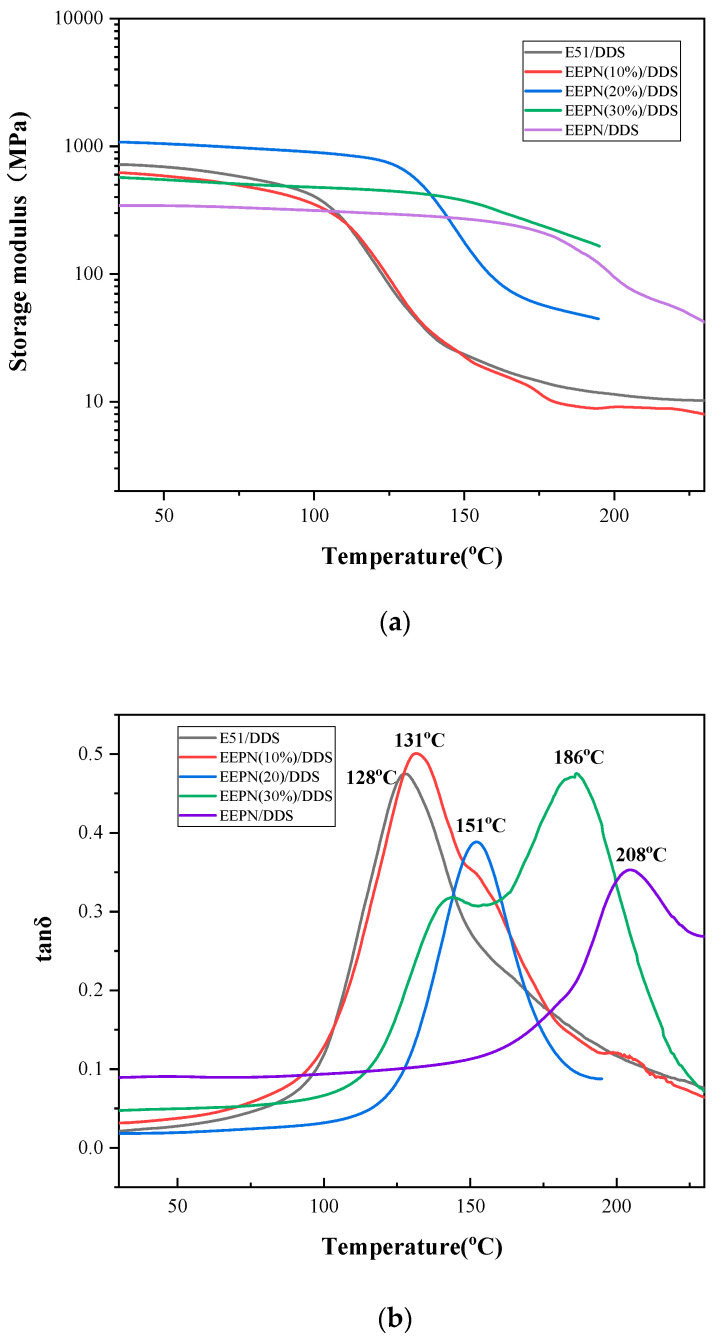
(**a**) Storage modulus and (**b**) tanδ for E51/DDS and EEPN(n)/DDS cured resins.

Figure 10 shows the DMTA test curves for cured E51/DDS and EEPN(n)/DDS. The storage modulus–temperature curves are shown in Figure 10a, and the loss factor–temperature curves are shown in Figure 10b.

According to Figure 10a, the initial energy storage modulus decreased with the increase in the EEPN addition, but the highest initial modulus of 1087 MPa was obtained for EEPN (20%)/DDS. The reason may be that after the introduction of a small amount of EEPN, the cross-linking point in the low-temperature curing interval (below 200 °C) decreased, which leads to a decrease in the modulus; after epoxy curing, the CN groups were restricted by the network formed by epoxy groups, which made it difficult to react sufficiently, thus leading to a decrease in the total cross-linking density and a decrease in the modulus. DDS can react with E51 to produce hydroxyl, which could catalyze CN cross-linking. When EEPN was added at 20%, a large number of triazine rings and isoindoline structures were formed, catalyzed by the hydroxyl group, and due to the small amount of EEPN added, there was less restriction on the epoxy network, which resulted in the largest modulus of EEPN (20%)/DDS.

The glass transition temperature (*T_g_*) of the cured material can be obtained from the curve of the loss factor tanδ versus temperature, as shown in Figure 10b. From the data in the figure, it can be seen that the *T_g_* of the EEPN (n)/DDS cured materials was higher than that of E51/DDS due to the high temperature-resistant cross-linking structure in the EEPN (n)/DDS cured materials, and the *T_g_* increased with the increase in the EEPN content, which suggested that introduction of the cyano structure in the epoxy resins could improve the heat resistance of the epoxy resins.

## 3. Materials and Methods

### 3.1. Materials

All the reagents were of analytical grade and used without further purification. N, N-Dimethylformamide (DMF) was obtained from Beijing Chemical Plant. (Beijing, China). Sodium hydroxide, perchloric acid, anhydrous ethanol, and dichloromethane were obtained from Beijing Tongguang Fine Chemical Company. (Beijing, China). Potassium hydrogen phthalate was purchased from J&K SCIENTIFIC. (Beijing, China). Deuterated chloroform (CDCl_3_), dimethyl sulfoxide (DMSO-d_6_), E51 epoxy resin, and 4,4′-diaminodiphenyl sulfones were purchased from Shanghai Aladdin Biochemical Technology Co., Ltd. (Shanghai, China). The 4-Nitrophthalonitrile, eugenol, anhydrous potassium carbonate, 3-chloroperoxybenzoic acid, crystalline violet, and tetraethylammonium bromide were purchased from Shanghai Macklin Biochemical Co., Ltd. In addition, EPN was synthesized according to the literature of Wang et al. [39] and Ning, Yi, et al. [41]. The FTIR spectra (KBr) (Figure S1), ^1^H NMR spectrum of EPN (a) and ^1^H spectrum of EPN in the range 6.87–8.03 ppm (b) (Figure S2), ^13^C NMR spectra (Figure S3), and elemental analysis of EPN (Table S1) are presented in the Supplementary Information.

### 3.2. Synthesis of EEPN

An amount of 0.01 mol (2.90 g) of eugenol-based *o*-phthalonitrile monomer with 0.02 mol (3.45 g) of 3-chloroperoxybenzoic acid was added to a 100 mL beaker with a magnetic stirring rotor. A quantity of 30 mL of dichloromethane was added as solvent, and the reaction was stirred at room temperature for 24 h. The dichloromethane was subsequently removed by heating, and the reaction product was added to 0.5 mol/L NaOH solution; the solid product was washed out and washed four times with deionized water until neutral. Subsequently, a filtration operation was carried out, and the product was dried in a vacuum oven at 60 °C for more than 6 h. A light-yellow solid powder, eugenol-based epoxy-phthalonitrile monomer, was finally obtained in a yield of 81.2%.

### 3.3. Determination of Epoxy Value

Based on the titration of perchloric acid glacial acetic acid solution using standard perchloric acid glacial acetic acid solution, crystal violet indicator, tetraethylammonium bromide, and potassium hydrogen phthalate (see Supplementary File), the data are shown in Tables S1 and S2. The concentration of standard perchloric acid glacial acetic acid solution was calculated to be 0.10195 mol/L, and the epoxide value of EEPN was 0.32.

### 3.4. Preparation of Epoxy Resins and Curing Process

#### 3.4.1. Preparation of Epoxy Resins

Bio-based epoxy resin EEPN was added to petroleum-based epoxy resin E51 in different proportions of the total mass of the resin to obtain different proportions of EEPN(n)/E51 hybrid resins. EEPN is a yellowish powdery substance at room temperature, and E51 is a colorless transparent viscous substance at room temperature. EEPN and E51 epoxy resins were accurately weighed in a beaker according to the mass ratios of 100:0, 30:70, 20:80, 10:90, and 0:100, and the specimens were labeled as EEPN, EEPN (30%)/E51, EEPN (20%)/E51, EEPN (10%)/E51, and E51 blends, respectively. The formulations of the blended resin and curing agent 4,4′-diaminodiphenyl sulfone (DDS) are shown in Table 4. The EEPN/E51/DDS mixture was dissolved in an appropriate amount of methylene chloride at room temperature and ultrasonically mixed well in a CNC ultrasonic cleaner, and then the solvent was removed by heating.

#### 3.4.2. Curing Process

**Figure 11:**
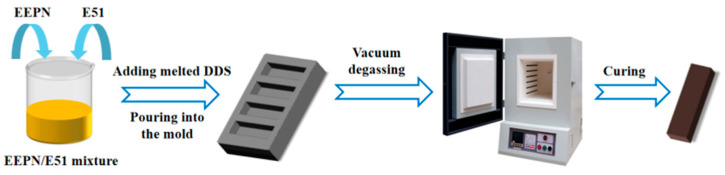
Schematic diagram of the curing process of EEPN/E51/DDS resins.

The EEPN/E51/DDS mixture was poured into the molds, and the curing process is shown in Figure 11. The curing was accomplished by undergoing a cure cycle of 150 °C for 2 h, 180 °C for 2 h, 210 °C for 2 h, 240 °C for 2 h, 300 °C for 2 h, and 330 °C for 2 h.

### 3.5. Characterization

The ^1^H and ^13^C NMR were measured on a Bruker ADVANCE 400 MHz spectrometer (Bruker Switzerland AG, Fällanden, Switzerland). Deuterated dimethyl sulfoxide (DMSO or CDCl_3_) was used as the solvent.

Fourier transform infrared (FTIR) spectra were recorded with a Nicolet-IS5 (Thermos Fisher Scientific, Waltham, MA, USA) spectrometer from 4000 to 400 cm^−1^ and 64 scans, using the KBr tablet preparation method for each sample.

Elemental analysis (EA) was performed using a Vairo EL CUBE type (GmbH, Mainhattan, Germany) elemental analyzer from 100% relative amounts of C, H, and N elements.

Differential scanning calorimetry (DSC) analysis was performed by a TA Instruments Q20 calorimeter under a nitrogen flow rate of 50 mL/min. Approximately 5 mg of each sample was sealed in aluminum pans, and the temperature was recorded between 25 and 350 °C at 10 °C/min, 5 °C/min, 3 °C/min, and 1 °C/min heating ramp.

Thermogravimetric analysis (TGA) was performed on a TGA Q500 (TA Instruments, New Castle, DE, USA) at a heating rate of 10 °C/min under a nitrogen purge of 100 mL/min. Approximately 10 mg of the specimen was placed in a ceramic crucible at a heating rate of 10 °C/min from 25 to 800 °C.

Dynamic mechanical thermal analysis (DMA) was performed using a Q800 (TA Instruments) dynamic mechanical analyzer in single-cantilever mode. DMA measurements were performed on cured rectangular specimens at 3065.5 mm^3^. The measurements were performed at a temperature range of 30–200 °C, a heating rate of 5 °C/min, and a frequency of 1 Hz, and the sample size was 10 × 6.0 × 1.5 mm.

## 4. Conclusions

A eugenol-based epoxy-phthalonitrile monomer (EEPN) was prepared via epoxidation reaction from a eugenol-based phthalonitrile monomer (EPN), and the latter was obtained from biobased eugenol, providing a concise method for the preparation of bio-based epoxy resins with outstanding properties of heat resistance. The curing process, curing mechanism, and curing reaction kinetics of EEPN/DDS were investigated using DDS as the curing agent. The heat-resistant modification effect of EEPN addition on conventional bisphenol A-type epoxy resin using DDS as curing agent was investigated by thermogravimetric analysis and dynamic thermomechanical analysis. Thermogravimetric analysis showed that with the increase in the EEPN content, the char yield of EEPN/E51 blended resin at 800 °C was gradually increased, and its heat resistance was significantly improved. The char yield of the EEPN cured product at 800 °C was 67.9 wt%, which was much higher than that of the E51 cured product at 26.3 wt%. In addition, the dynamic thermo-mechanical analysis showed that due to the high-temperature resistant cross-linking structure in the EEPN(n)/DDS cured resins, the *T_g_* of the EEPN(n)/DDS resins was higher than that of E51/DDS, with EEPN (100)/DDS having a *T_g_* of up to 208 °C, which was considerably higher than that of E51/DDS (128 °C). The results proved that the introduction of cyanide structure groups into the molecular structure of epoxy resins using the amine curing agent improved the property of heat resistance.

## Figures and Tables

**Table 1:** Elemental analysis result of EEPN.

Element	C (%)	H (%)	N (%)	C/H
Theoretical value	70.60	4.57	9.15	15.45
Actual value	69.69	4.43	9.34	15.71

**Table 2:** DSC data of E51/DDS and EEPN(n)/DDS resins.

Samples	*T_i_* (°C)	*T_p_* (°C)	*T_f_* (°C)	∆T (°C)
E51/DDS	162.8	211.5	254.7	91.9
EEPN (10%)/DDS	138.6	240.2	309.3	170.7
EEPN (20%)/DDS	146.9	241.4, 281.3	323.8	176.9
EEPN (30%)/DDS	158.3	241.9, 281.5	322.0	163.7

**Table 3:** TGA and DTG data of E51/DDS and EEPN(n)/DDS (N_2_).

Cured Samples	*T_d__5_* (°C)	*T_d10_* (°C)	*T_d30_* (°C)	*T_dmax_* (°C)	*Y_c800_* (%)	*T_s_*
E51/DDS	330.2	359.9	391.2	400.0	26.3	179.7
EEPN (10%)/DDS	342.4	364.2	392.9	395.7	26.8	182.6
EEPN (20%)/DDS	359.9	373.0	396.4	394.6	29.7	187.1
EEPN (30%)/DDS	363.4	373.7	398.2	388.4	38.2	188.3
EEPN/DDS	372.2	399.3	694.2	393.7	67.9	277.0

**Table 4:** The formulation of EEPN/E51/DDS resins.

Samples	EEPN	DDS
EEPN	100	25
EEPN (30%)/E51	100	28.5
EEPN (20%)/E51	100	29.0
EEPN (10%)/E51	100	29.5
E51	100	30

## Data Availability

Data presented in this study are available on request from the corresponding author.

## Supplementary Materials

The following supporting information can be downloaded at https://www.mdpi.com/article/10.3390/molecules29215019/s1: Figure S1: FTIR spectra of EPN and raw materials; Figure S2: ^1^H NMR spectrum of EPN (a) and ^1^H spectrum of EPN in the range 6.87–8.03 ppm (b); Figure S3: ^13^C NMR spectrum of EPN; Figure S4: ^1^H spectrum of EEPN in the range 6.97–8.04 ppm; Figure S5: **^1^**^3^C NMR spectrum of EEPN; Figure S6: The non-isothermal curing DSC curve of EEPN/DDS; Figure S7: Liner fitting curves of EEPN/DDS resins: (a) Ozawa equation and (b) Kissinger equation; Table S1: Elemental analysis results of EPN; Table S2: Results of acid calibration; Table S3: Epoxy titration results of EEPN; Table S4: The non-isothermal DSC data from the curing process of EEPN/DDS; Table S5: The parameters of non-isothermal DSC for EEPN/DDS; Table S6: Parameters of curing kinetics.
