# Supplementary material for: Bio-Based Epoxy-Phthalonitrile Resin: Preparation, Characterization, and Properties
Source: Molecules. 2024 Oct 24;29(21):5019. doi: 10.3390/molecules29215019 (PMC11547601; doi:10.3390/molecules29215019)
Supplement: Supplementary file 1 [file molecules-29-05019-s001.zip › molecules-3180954-supplementary.pdf]

Supporting Information

Bio-based epoxy-phthalonitrile resin: preparation, characterization and properties

Yanqin Du [1], Ruojin Wang [1], Qingxu Meng [1], Xiaoa Zhang [1,*] and Riwei Xu [1,*]

1. College of Materials Science and Engineering, Beijing University of Chemical Technology, Beijing 100029, China; d1584946123@163.com (Y. D.); ruojin1208@gmail.com (R. W.); mqx2091093720@outlook.com (Q. M.); zhangxiaoa1982@163.com (X. Z.); xurw@mail.buct.edu.cn (R. X.)
*Correspondence: xurw@mail.buct.edu.cn (R. X.); zhangxiaoa1982@163.com (X. Z.)

Synthesis of EPN

An eugenol-based phthalonitrile monomer was prepared from eugenol and 4-nitrophthalonitrile by a nucleophilic substitution reaction catalysed by K_2CO_3 in the process shown in Scheme 1[1,2]. 0.1 mol of eugenol (16.420 g), 0.1 m ol of 4-nitrophthalonitrile (17.313 g) and 0.12 mol of anhydrous potassium car bonate (16.585 g) were added together in a 500 ml single-necked flask along with 250 mL of DMF as solvent and equipped with a magnetic stirring rotor. The temperature was raised to 80°C in a thermostatic oil bath and kept for 8 h reaction time, followed by cooling to room temperature. The solvent DMF w as removed by evaporation at 80°C using a rotary evaporator, and the reaction solution was alkaline washed in 0.1 mol/L NaOH solution, washed four times with deionized water to neutrality, and then subjected to a filtration process. Then it was dried in a vacuum oven at 60°C for more than 6 h. The product was a light brown solid powder with a yield of 58.8%.

Materials and Methods

Materials are listed in the section of Materials and methods in the article.

^{1}H and ^{13}C NMR were measured on a Bruker ADVANCE 400 MHz spectrometer(Bruker Switzerland AG). Deuterated dimethyl sulfoxide (DMSO or $CDCl_3$) was used as the solvent.

Fourier transform infrared (FTIR) spectra were recorded with a Nicolet-IS5 (Thermos Fisher Scientific)spectrometer from 4000 to 400 cm^{-1} and 64 scans, using the KBr tablet preparation method for each sample.

Elemental analysis (EA): Vairo EL CUBE type (GmbH, Germany) elemental analyzer from, 100% relative amounts of C, H and N elements.

Differential scanning calorimetry (DSC) analysis was performed by a TA Instruments Q20 calorimeter under a nitrogen flow rate of 50 mL/min. Approximately 5 mg of each sample was sealed in aluminum pans, and the temperature was recorded between 25 and 350°C at 10°C/min, 5°C/min, 3°C/min and 1°C/min heating ramp.

1. Characaterization of eugenol-based phthalonitrile monomer (EPN)

From Figure S1, it can be seen that the hydroxyl stretching vibration peak at 3514 cm^{-1} in the eugenol monomer disappeared in EPN and the cyano stretching vibration peak at 2228 cm^{-1} in the 4-nitrophthalonitrile monomer appeared in the product EPN, which indicates that the hydroxyl group and the cyano group have been fully reacted. At the same time, new peaks appeared in the product EPN. The absorption bands in all the monomers were recorded at 1244 cm^{-1} as the asymmetric stretching vibrations of the C-O-C, while symmetric stretching vibrations of the C-O-C appeared at 1042 cm^{-1}. EPN has a C=C stretching vibration at 1638 cm^{-1} while the absorption bands at 3075 cm^{-1}, 998 cm^{-1} and 913 cm^{-1} belong to the -C=H stretching vibration of allyl. Therefore, it can be determined that EPN was successfully synthesized.

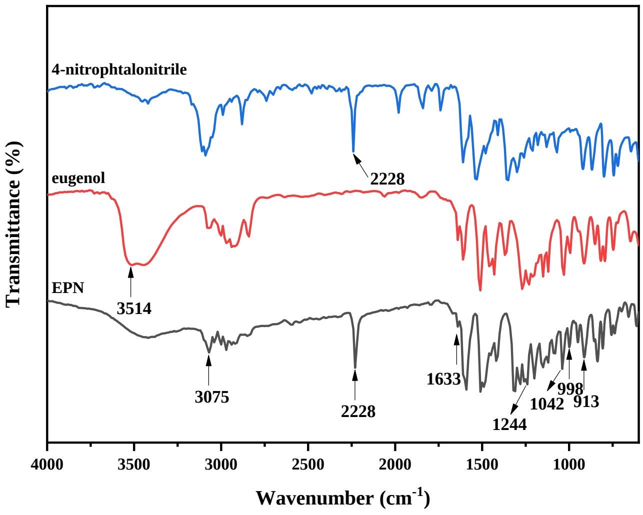

Figure S1 FTIR spectra of EPN and raw materials.

Figure S2(a) shows the ^1H NMR spectrum of EPN, which has 10 differen t chemical shifts of H. From the figure S2, it can be seen that δ=3.32 ppm is the characteristic absorption peak of H on H_2O, and 2.50 ppm is the characte ristic absorption peak of H on the deuterium solvent DMSO. δ=5.11 ppm, δ=6. 01 ppm, and δ=3.43 ppm are the characteristic peaks of H_a, H_b, and H_c on all yl groups, respectively. Their integral area ratios are in accordance with the th eoretical ratio of 2:1:2. The benzene ring structure is a spatially asymmetric th

ree-dimensional structure, so its hydrogen atoms are in different chemical environments, and the aromatic protons of the spectra are confirmed at 6.84-7.78 ppm. We zoomed in the range of 6.84-7.78 ppm, from Fig.S2(b). It can be seen that H_d is a single peak, but by H_e's interstitial smaller coupling effect appeared small crack, H_e and H_j mutual coupling is cracked into double peaks. At the same time, H_e by the interstitial H_d coupling effect, appeared the second level of cracked peaks. Similarly, in the H_g, H_h and H_i there is also such a mutual coupling of the situation. The details are as follows: H_d(s, δ=7.08ppm), H_e (d, δ=6.88-6.86ppm) H_g(s, δ=7.66ppm), H_h(dd, δ=7.20-7.18ppm) H_i(d, δ=8.04-8.02ppm), and H_j(d, δ=7.15-7.13ppm), the integral areas of H_d, H_e, H_g, H_h, H_i, and H_j, belong to 1:1:1:1:1:1. In summary, by comparison with the published literature of Wang et al. it can be demonstrated that EPN was successfully synthesized.

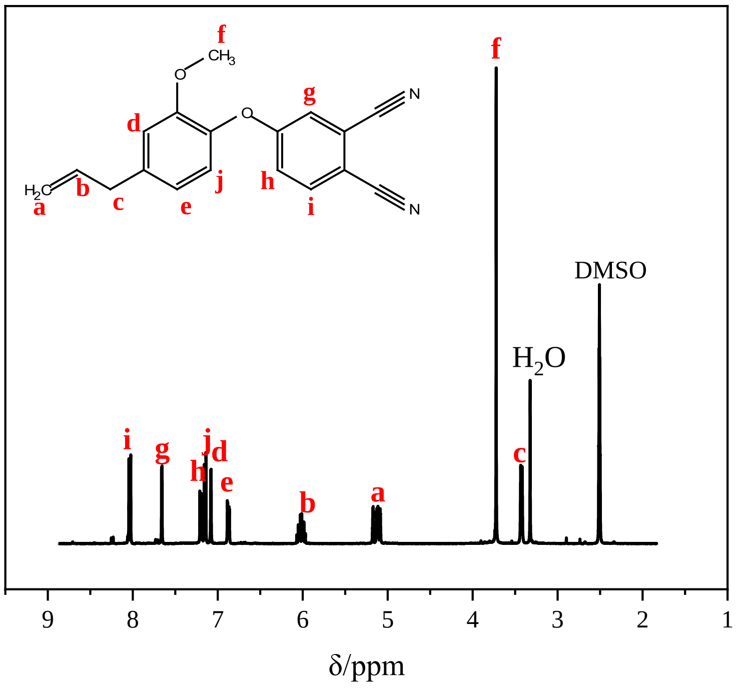

(a)

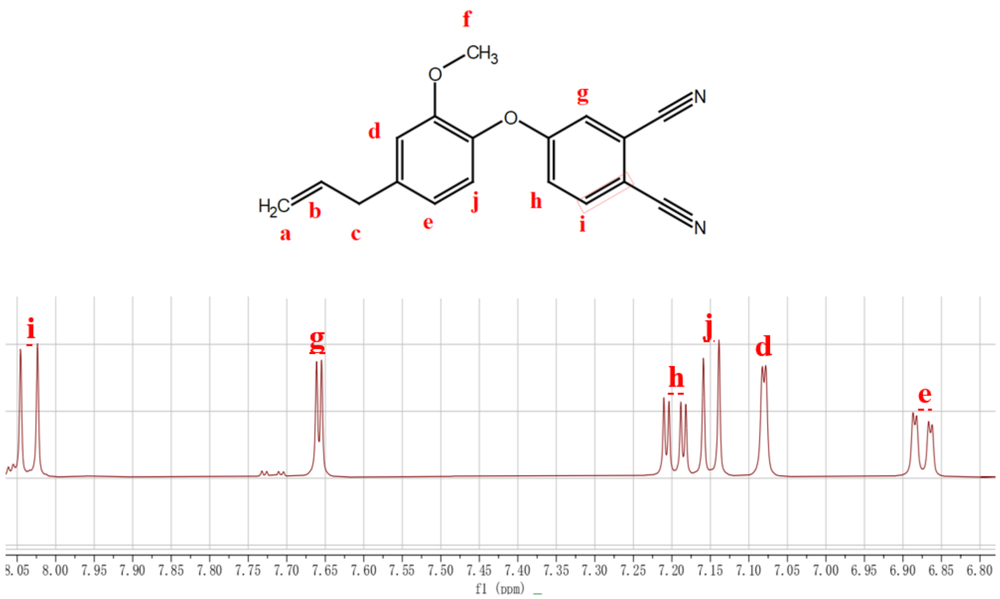

(b)

Figure S2 ^{1}H NMR spectrum of EPN(a) and ^{1}H spectrum of EPN in the range 6.87-8.03 ppm(b).

Figure S3 shows the ^{13}C NMR spectrum of EPN, from which it can be seen that methylene carbon atom features appear at δ=40.07 ppm, δ=55.61 ppm for the characteristic peaks of methoxy on the benzene ring, δ=116.54 ppm and 136.68 ppm for the characteristic peaks of $=CH_2$ and =CH-, and δ=117.3 ppm, δ=116.6 ppm for the characteristic peaks of the two characteristic peaks for the cyano group. The chemical shifts of the carbon atoms remaining on the benzene ring are shown in Fig. 5. (C_a: δ=113.43 ppm, C_b: δ=136.18 ppm, C_c: δ=123.98, C_d: δ=120.5 ppm, C_e: δ=140.05 ppm, C_f: δ=151.07 ppm, C_g: δ=161.99 ppm, C_h: δ=122.35 ppm, C_i: δ=135.18 ppm, C_j: δ=108.19 ppm, C_k: δ=115.65ppm.)

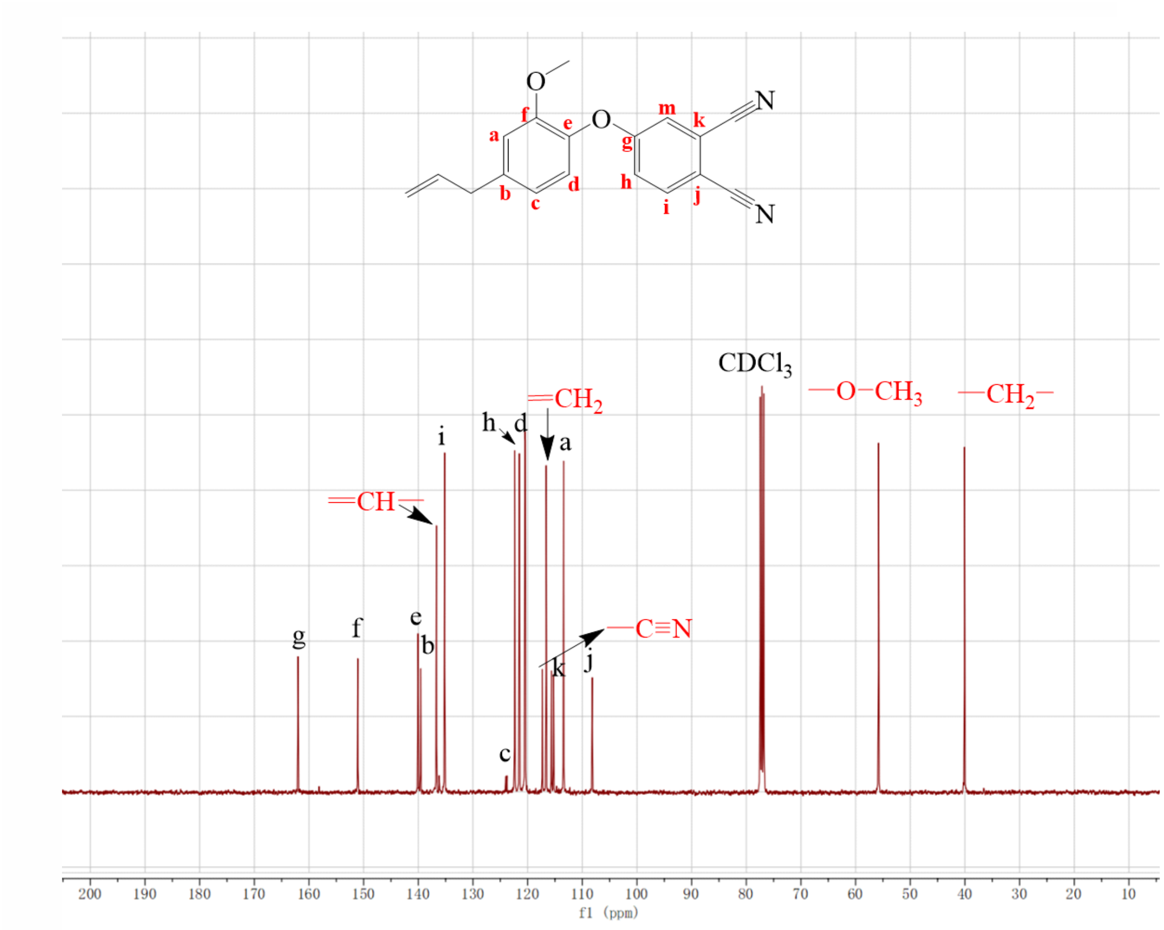

Figure S3 ^{13}C NMR spectrum of EPN.

Table S1 shows the results of elemental analysis of EPN, which has the molecular formula of $C_{18}H_{14}N_2O_2$ and molecular weight of 290.32. The theoretical contents of C, H and N in EPN are 74.48%, 4.83% and 9.66%, respectively, with a theoretical carbon-hydrogen ratio of 15.42; and the actual contents of C, H and N in the product are 73.71%, 4.73% and 10.04%, with a theoretical the deviation of C, H and N contents in the product EPN was within the error range, indicating that the synthesized EPN was as expected.

Table S1 Elemental analysis results of EPN.

element	C%	H%	N%	C/H
theoretical value	74.48	4.83	9.66	15.42
actual value	73.71	4.73	10.04	15.59

2. NMR spectrum of EEPN and its explanation

The NMR distribution of EEPN aromatic proton hydrogens is supplemented here. For the distribution of aromatic protons of EEPN as shown below, we re-tested and checked. In the following figure 4, we enlarged the ^{1}H spectrum of EEPN in the range of 6.97-8.04ppm part, it can be seen that H_e and H_j are coupled to each other and split into double peaks, at the same time, H_e is coupled by the interstitial H_d, and there is a secondary split peak, H_h is coupled by the neighbouring H_i and splits into double peaks, and at the same time, it is coupled by the smaller interstitial of H_g, and finally shows the morphology of the approximate quadruple peaks, and there is also a case of such a mutual coupling between H_g and H_i and so there is a double peak. The details are as follows: H_d(s, δ=7.16ppm), H_e(d, δ=6.88-6.86ppm) H_g(s, δ=7.69ppm), H_h(dd, δ=7.21-7.19ppm) H_i(d, δ=8.04-8.02ppm), 和 H_j(d, δ=7.18-7.17ppm), and the ratio of their integral area is 1:1:1:1:1:1, proving that the EEPN is successful in the basis of the EPN combined out.

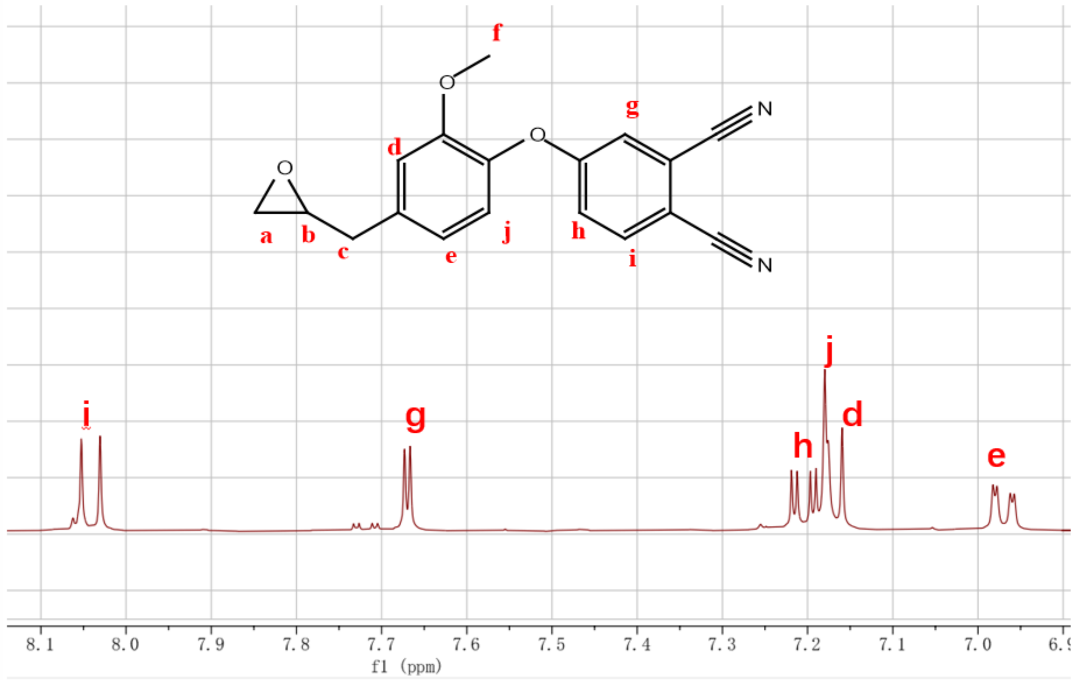

Figure S4 ^{1}H spectrum of EEPN in the range 6.97-8.04 ppm.

The following figure shows the ^{13}C NMR spectrum of EEPN with dmso as solvent, from which it can be seen that δ=116.45 ppm and δ=116.92 ppm are the characteristic peaks of cyano group, δ=56.22 ppm are the characteristic peaks of C_j for methoxy on the benzene ring, δ=46.65 ppm and 52.31 ppm are the characteristic

peaks of C_a and C_b for epoxy group, and δ=38.41 ppm is the characteristic peak of C_c the characteristic peak of C_c. The distribution of aromatic carbon atoms was as follows, C_e: δ=114.86 ppm; C_d: δ=137.82 ppm; C_f: δ=122.22 ppm; C_g: δ=120.64 ppm; C_h: δ=139.95 ppm; C_i: δ=151.22 ppm; C_k: δ=161.89 ppm; C_l: δ=121.57 ppm; C_m: δ=136.6 ppm; C_n: δ=107.87 ppm; C_o: δ=115.93 ppm; C_p: δ=122.69 ppm.

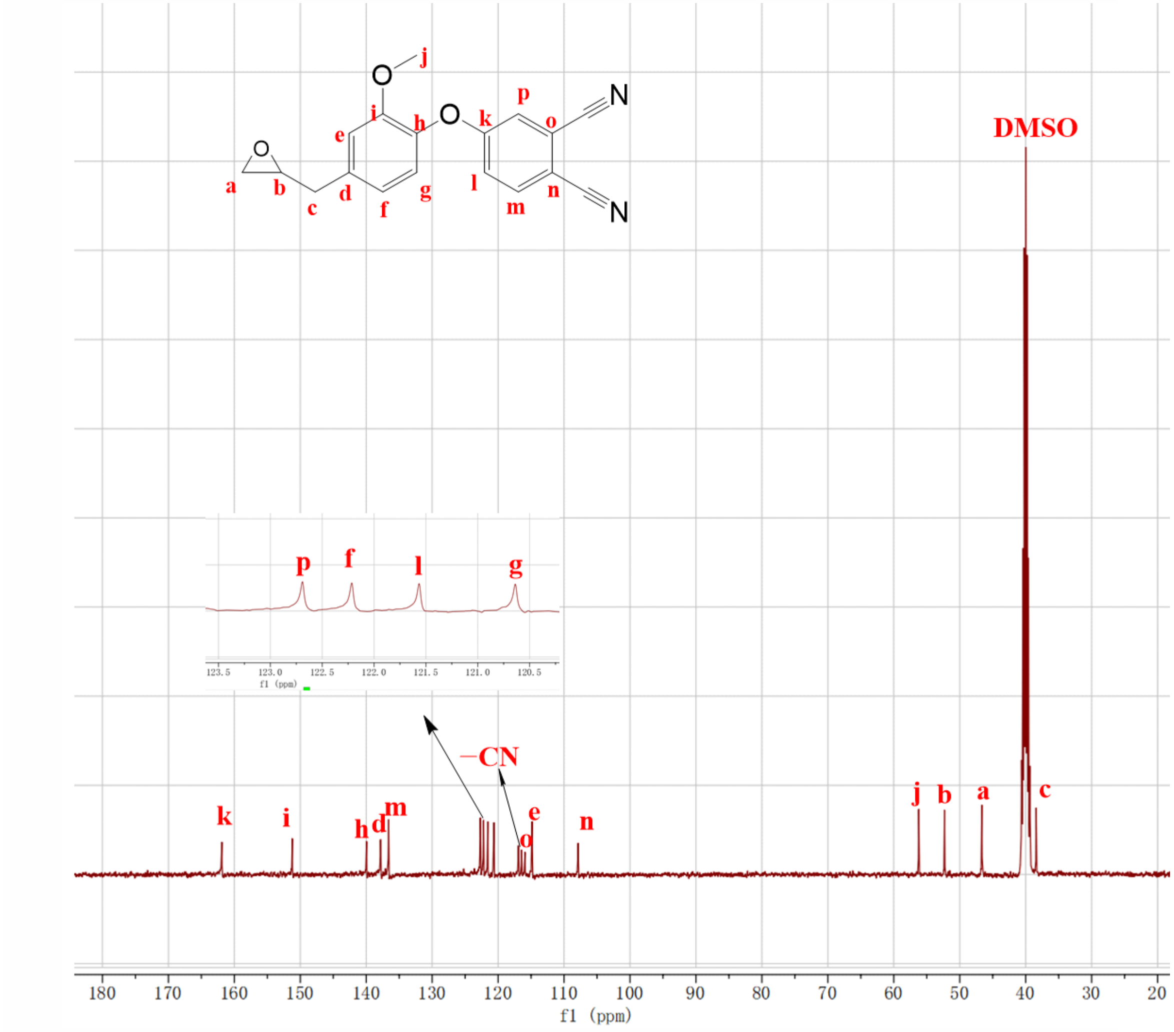

Figure S5 ^{13}C NMR spectrum of EEPN.

3. Determination of epoxy value

Calibration of standard solution: Measure 300 ul of 70% perchloric acid and add it to 30 ml of glacial acetic acid, shake and continue to add glacial acetic acid to a total volume of 40 ml, which is prepared as perchloric acid standard solution. Weigh 10 mg of crystal violet solid into 10 ml of glacial acetic acid, shaking to dissolve, the solution showed a deep purple color, prepared as crystal violet - acetic acid solution.

A tetraethylammonium bromide solution was prepared by weighing 4 g of tetraethylammonium bromide solid into 20 ml of glacial acetic acid and shaking to dissolve. Weigh about 0.2 g of potassium hydrogen phthalate into a beaker, add about 20 ml of glacial acetic acid to make it dissolve, add 2 drops of crystal violet indicator, titrate with the perchloric acid glacial acetic acid solution to be calibrated, the end point of the titration when the solution turns from violet to green, and record the volume of the perchloric acid acetic acid solution used.

Titration of epoxy value: weigh 0.1 g of the product EEPN, dissolve it in 5 ml of glacial acetic acid and put it into a 50 ml beaker. It was stirred at room temperature. To it 2.5 ml of acetic acid solution of tetraethylammonium bromide was added and 3 drops of crystal violet indicator was added to it and the solution showed blue-violet color. Calibration was carried out using perchloric acid glacial acetic acid standard solution. The color of the solution changes from violet to blue to green during the titration and the end point of the titration is a stable green color.

Table S2 Results of acid calibration

potassium hydrogen phthalate (g)	perchloric acid/glacial acetic acid solution（mL)	c (mol/L)
0.203	9.7	0.1035
0.203	9.8	0.1014

Table S3 Epoxy titration results of EEPN

EEPN(g)	perchloric acid glacial acetic acid solution（mL)	Epoxy value
0.103	1.15	34.84%
0.100	1.05	31.21%
0.102	1.10	32.12%

4. Calculations for Curing kinetics

The apparent activation energy (E) of resins could be calculated by the Kissinger (Equation (1)) and Ozawa (Equation (2)) methods. The number of reaction stages n was calculated by Ozawa's method as in equation (3).Calculate the prefinger factor A

from the Arrhenius formula, the mean value of activation energy E, as in equation (4)

T_p is the peak exothermic temperature in the DSC curve; β is the heating rate; Ea is the apparent activation energy; R is the molar gas constant, 8.314 J/(mol K); n is the number of curing reaction stages.

$$\frac{E_a}{R} = -\frac{d\ln(\beta/T_p^2)}{d1/T_p} \tag{1}$$

$$\ln\beta = -1.052\frac{E_a}{RT_p} \tag{2}$$

$$\frac{d\ln\beta}{d1/T_p} = -\left[\frac{E_a}{nR} + 2T_p\right] \tag{3}$$

$$\mathrm{A} = \frac{\beta E exp(\frac{E}{RT_p})}{RT_p^2} \tag{4}$$

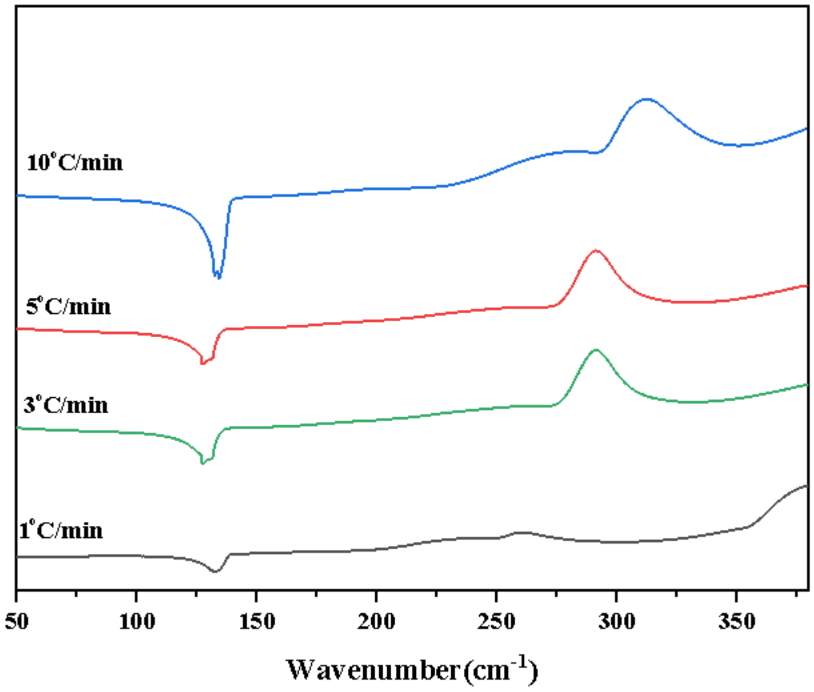

Figure S6 The non-isothermal curing DSC curve of EEPN/DDS.

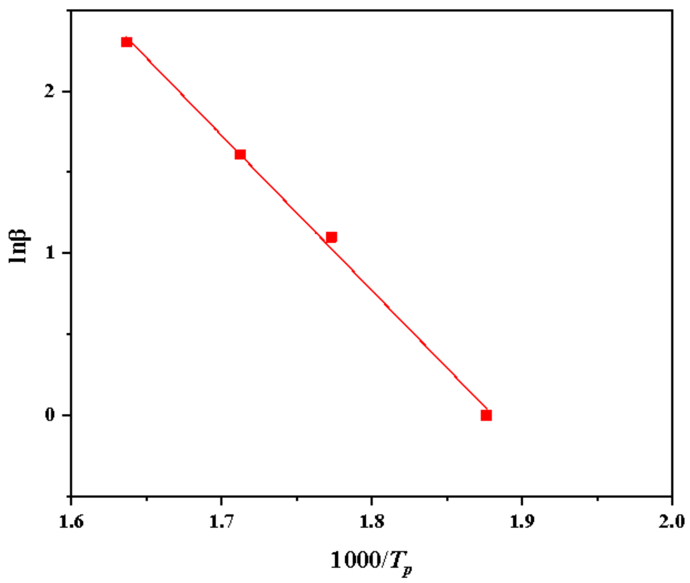

(a)

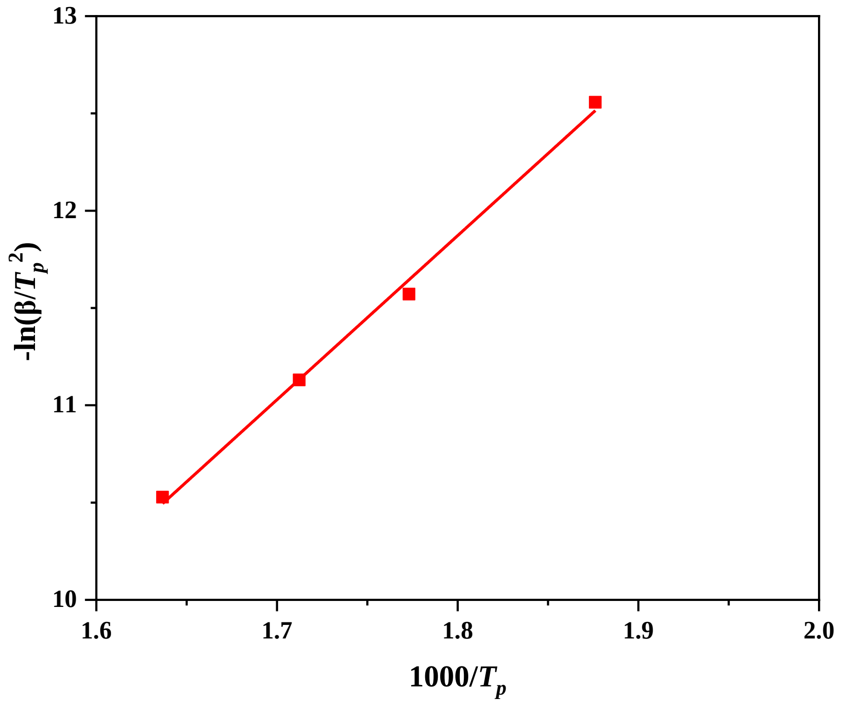

(b)

Figure S7 Liner fitting curves of EEPN/DDS resins: (a) Ozawa equation; (b) Kissinger equation.

Table S4 The non-isothermal DSC data from curing process of EEPN/DDS

samples	β (°C/min)	T_i(°C)	T_p(°C)	T_f(°C)	ΔT(°C)	-ΔH(J/g)
EEPN/DDS	1	202	260	300	98	101.4
	3	216	291	319	103	106.2
	5	226	311	345	119	100.2
	10	253	338	368	115	73.8

Table S5 The parameters of non-isothermal DSC for EEPN/DDS

samples	β/(°C/min)	T_p/K	$-\ln(\beta/T_p^2)$	$1000/T_p$(K^{-1})	lnβ
EEPN/DDS	1	533	1.88	12.56	0
	3	564	1.77	11.57	1.10
	5	584	1.71	11.13	1.61
	10	611	1.64	10.53	2.30

Table S6 Parameters of curing kinetics

samples	β/(°C/min)	n	A
EEPN/DDS	1	0.87	2.55×10^7
	3	0.95	2.77×10^7
	5	1.03	2.53×10^7
	10	0.99	2.38×10^7
	average value	0.96	2.56×10^7

References:

1. Wang, A. R., Dayo, A. Q., Zu, L. W., Lv, D., Song, S., Tang, T., Gao, B. C. Bio-based phthalonitrile compounds: Synthesis, curing behavior, thermomechanical and thermal properties. *React Funct Polym* **2018**, 127, 1-9.

2. Ning, Y., Li, D. S., Wang, M. C., Jiang, L. Bio-resourced eugenol derived phthalonitrile resin for high temperature composite. *J. Appl. Polym* **2021**, 138, 50721.
